# Why People Take Medicine

**Published:** 1885-02

**Authors:** 


### WHY PEOPLE TAKE MEDICINE.

In the *British Quarterly Review*, a prominent authority (Dr. Crofts) thus speaks on this point :

"It is to be feared that, to most people, medicine is not an erudite science, or a learned art, but is little more than the commonplace administration of physic. They cannot understand medicine without drugs, and its virtue and power are popularly measured by the violence of its operations. Its very name is, in ordinary parlance, synonymous with physic. Take from it its pills and potions, and for them you take away its whole art and mystery. They do not believe in a scheme of treatment, however deep-laid and skillful, which does not include a certain statutory dosage; so that, as a rule, medical men are practically compelled to give their patients a visible object of faith in some form of physic, which may be, at most, designed to affect some very subordinate purpose. And it is remarkable how strongly, even among the educated classes, this feeling prevails. Cure by the administration of mixtures and boluses is so fixed and ancient a tradition, that it is only very slowly that the world will give it up. The anxiety of the friends of the patient wants to do more than follow the simple directions of 'nursing,' which have been so carefully inculcated, and possess, apparently, so little remedial power. There is nothing of the unknown about them in which a fluttering hope of great advantage can nestle. Thus it is necessary to educate the world into a belief in medicine, apart from drugs, which finds its power of curing in adaptation of the common conditions of life and applications of physiological facts—a medicine which takes into its hands the whole life, and orders and fashions, in every detail, with scientific definiteness. It is found in every-day practice that this popular misunderstanding of the modern spirit of medicine constantly checks the little tentative advances of a more scientific treatment, and it is necessary that it should be generally understood how powerfully the various processes of the economy may be affected by the manipulation of the conditions of common life."

